# Oxygen-dependent laser inactivation of murine norovirus using visible light lasers

**DOI:** 10.1186/s12985-018-1019-2

**Published:** 2018-07-31

**Authors:** David Kingsley, Robinson Kuis, Rafael Perez, Isaac Basaldua, Paul Burkins, Aristides Marcano, Anthony Johnson

**Affiliations:** 1 USDA ARS ERRC Food Safety Intervention Technologies Research Unit, Dover, Delaware USA; 2 Center for Advanced Studies in Photonics Research, University of Maryland Baltimore County, Baltimore, USA; 3 Department of Physics and Engineering, Delaware State University, Dover, Delaware USA; 4 Department of Physics, University of Maryland Baltimore County, Baltimore, USA; 5 Department of Computer Science and Electrical Engineering, University of Maryland Baltimore County, Baltimore, USA

**Keywords:** Blue light, Singlet oxygen, Virus

## Abstract

**Background:**

Previous work indicated that an ultrashort pulse (USP) 425 nm laser is capable of inactivating murine norovirus (MNV: Virol. J. 11:20), perhaps via an impulsive stimulated Raman scattering (ISRS) mechanism, and does not substantially damage human plasma proteins (PLOS One 9:11). Here, further investigation of virus inactivation by laser light is performed.

**Methods:**

In this study, we evaluate whether inactivation of MNV is specific to the USP wavelength of 425 nm, or if it occurs at other visible wavelengths, using a tunable mode-locked Ti-Sapphire laser that has been frequency doubled to generate femtosecond pulses at wavelengths of 400, 408, 425, 450, 465, and 510 nm. Continuous Wave (CW) lasers are also applied. Singlet oxygen enhancers are used to evaluate the sensitivity of MNV to singlet oxygen and oxygen quenchers are used to evaluate effects on virus inactivation as compared to untreated controls.

**Results:**

> 3 log_10_ inactivation of MNV pfu occurs after irradiation with an average power of 150 mW at wavelengths of 408, 425 or 450 nm femtosecond-pulsed light for 3 h. Thus results suggest that the mechanism by which a laser inactivates the virus is not wavelength-specific. Furthermore, we also show that irradiation using a continuous wave (CW) laser of similar power at 408 nm also yields substantial MNV inactivation indicating that inactivation does not require a USP. Use of photosensitizers, riboflavin, rose bengal and methylene blue that generate singlet oxygen substantially improves the efficiency of the inactivation. The results indicate a photochemical mechanism of the laser-induced inactivation where the action of relatively low power blue laser light generates singlet oxygen.

**Conclusion:**

Results suggest formation of short-lived reactive oxygen species such as singlet oxygen by visible laser light as the cause of virus inactivation rather than via an ISRS mechanism which induces resonant vibrations.

## Background

Human noroviruses are the most prevalent cause of food-borne illness in the US with an estimated overall infection rate that may be as high as one in six persons annually [36]. These viruses are known to be highly resilient, persisting in the environment and foods for periods of a month or more [18]. Nonthermal intervention options that are able to inactivate these noroviruses and other enteric viruses in foods and pharmaceuticals are limited. Sunlight has been implicated as a means by which viruses are environmentally inactivated [10, 11, 14, 21, 22, 37, 38]. Indeed human norovirus was first known as the winter vomiting disease since its person-to-person transmission peaks during fall and winter months [13, 52] and its elevated presence in sewage and shellfish is typically observed in the winter season [4].

Atmospheric sunlight at sea level contains visible light (400–700 nm) but also contains a UV component, that is predominately 300–400 nm [3, 12, 25] which does not substantially penetrate water since UV is strongly absorbed. Recent research has indicated that sunlight can interact with dissolved organic molecules in aqueous settings to catalyze formation of O_2_ (a^1^∆_g_), commonly referred to as singlet oxygen, as well as other reactive oxygen species [7, 9, 22, 35]. These complex organic molecules act as sensitizers that become energetically excited by UV–Vis light and then transfer electronic energy to oxygen molecules producing singlet oxygen. In fact this basic mechanism, by which light interacts with sensitizer molecules, is commonly utilized to inactivate enveloped viruses within plasma and blood products [5, 6, 8, 23, 29–31, 34, 40]. It has also been shown that blue (405 nm) light can inactivate bacteria via its interactions with porphyrins (5 and 6 carbon multi-ring contain alternating single and double bonds) within the membrane of bacteria [28], and is actively being investigated as a means of disinfection of medical and food surfaces ([2, 17, 19, 27, 39]). Complex compounds such as methylene blue, rose bengal (a.k.a. red food dye 105) and even riboflavin (a.k.a vitamin B2) can also function as sensitizers to produce singlet oxygen [15, 24, 50].

Ultrashort pulse laser (USPL) light treatments were previously shown to be capable of inactivating murine norovirus (MNV) and other viruses [42, 45, 47–49]. Impulsive stimulated Raman scattering (ISRS) was the postulated inactivation mechanism [43, 44, 46]. Essentially the ISRS hypothesis was that high frequency resonance vibrations are potentially induced by the 425 nm USPL, with a bandwidth of 420–430 nm, that may be capable of causing vibrations of sufficient strength that the capsid is destroyed after nonthermal treatments of an hr. or more [43]. Here we evaluate whether the mechanism of laser-induced virus inactivation is strictly wavelength-dependent and pulse width-dependent using a tunable Ti:Sapphire USPL, having an 76 MHz repetition rate and a pulse width of 120 fs, and a continuous wave (CW) 408 nm wavelength laser.

## Methods

### Murine norovirus

Working stocks of MNV were prepared using confluent RAW264.7 cells propagated in high glucose Dulbecco’s modifies Eagle media with 25 mM hepes buffer without indicator dye. Four days post-infected monolayers were freeze/thawed, clarified by centrifugation and 0.2 μm filtered. MNV stock titers ranged from 0.5 × 10^5^ to 1 × 10^7^ pfu/ml. For experiments with singlet oxygen enhancers, rose bengal (Sigma-Aldrich, St. Louis MO) and riboflavin (Sigma-Aldrich) were then added to MNV at a final concentration of 0.1%. For oxygen depletion experiments, MNV stock was supplemented with 200 and 1000 ppm sodium bisulfite (Sigma-Aldrich).

Following treatments, samples were assayed using confluent 6-well dishes inoculated with 0.5 ml or 10-fold serial dilutions prepared in Earle’s balanced salt solution (EBSS; Life Sciences) for 2 h at 37 °C followed by overlay with 2 ml of modified Eagle media (Gibco-Invitrogen) containing 1.5% low melt agarose (Fisher Biotech) with 5% FBS, 2 mM Gluta-MAX-1 (Gibco-Invitrogen), 100 U of penicillin and 100 μg/ml of streptomycin sulfate (Gibco-Invitrogen). After 3–4 days, plaques were visualized by staining with 0.03% neutral red (Fisher Biotech) for 2–4 h at 37 °C.

### Laser treatments

**Fig. 1:**
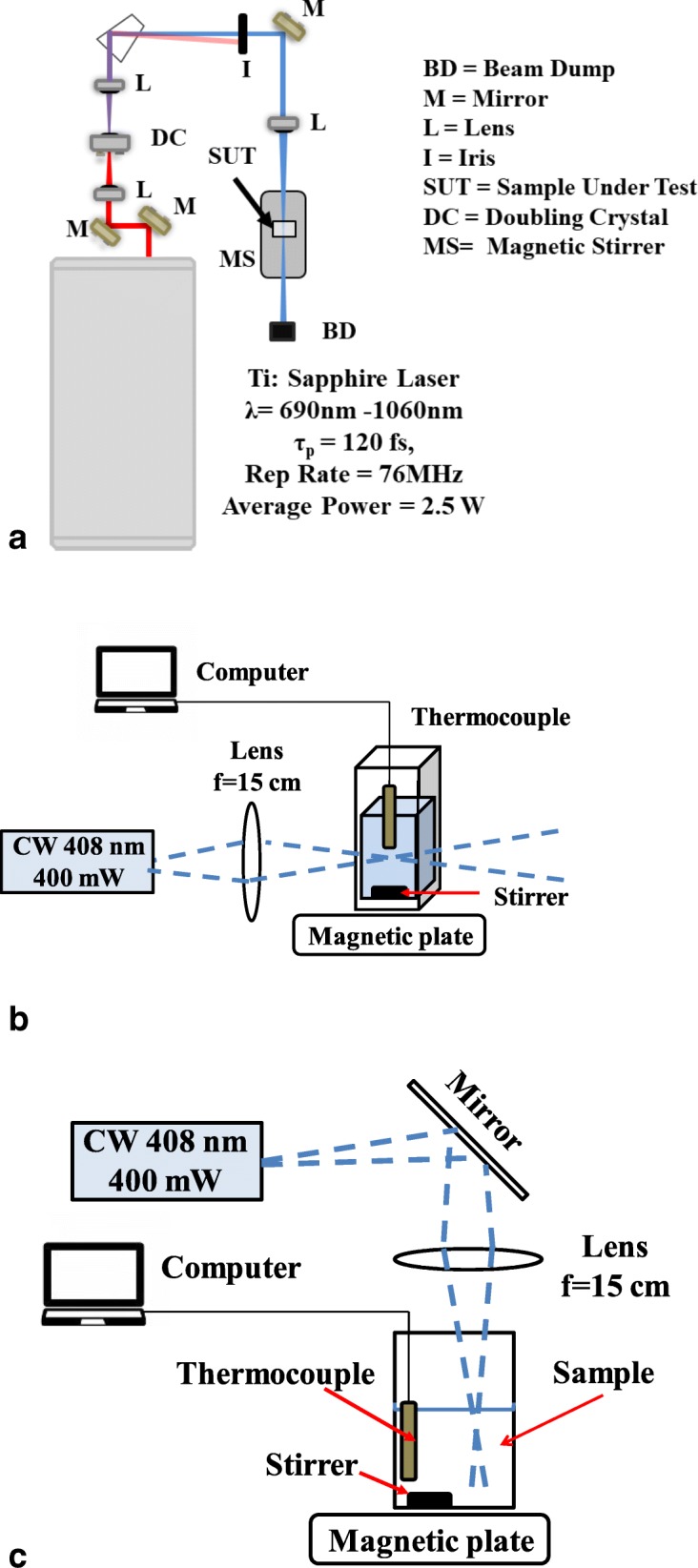
Laser virus inactivation experimental set-ups **a**) USP laser in vertical configuration **b**) Continuous wave 408 nm in horizontal configuration **c**) Continuous wave 408 nm in vertical configuration

A schematic of the USPL set-up is shown in Fig. 1a. The Ti: Sapphire laser (Spectra-Physics Mai Tai, Santa Clara, CA) is pumped by a 10 W 532 nm Neodymium Vanadate laser which was frequency doubled from 1064 nm. The USPL generates 120-fs at a repetition rate of 76 MHz- and tunable wavelengths between 690 and 1060 nm. The light was frequency doubled (BBO crystal 6 × 6 × 0.7 mm, Altos Photonics, Inc., Bozeman, MT) to select wavelengths of 400, 408, 425, 450, and 465 nm. A 1-cm path length quartz cuvettes containing 1–2 ml of MNV sample were irradiated for various time durations (from zero to 4 h) and power levels (from 40 mW to 400 mW). A 15-cm lens focused the excitation light onto the sample resulting in pulse intensities in the range of 0.98 to 9.8 GW/cm^2^. A small spin bar (8 mm × 3 mm) and a magnetic stirrer were used to agitate the samples during treatment within the quartz cuvette. Experiments were also conducted using a 10 W 10 ps Neodymium Vanadate laser (Lumentum, 400 North McCarthy Blvd, Milpitas, CA) operating at a wavelength of 1064 nm with a repetiton rate of 76 MHz. The 10 ps laser was frequency doubled to 532 nm using a potassium titanyl phosphate (KTP) crystal prior to MNV treatments. The frequency-doubled laser produced 7 ps pulses at 532 nm and 1 W of average power. A 15-cm focal-length lens focused this light onto the sample resulting in a pulse intensity of 0.26 GW/cm^2^. Continuous wave laser treatments of MNV were performed using a CW multi-mode diode laser at 408 nm wavelength (Lasever Inc., Ningbo, China). Depictions of the two different set-ups for the 408 nm CW laser are shown in Fig. 1b and c. A 15-cm focal-length lens focused the light onto the sample resulting in an average intensity of 39 KW/cm^2^. For 408CW defocused experiments CW (Fig. 1c), the beam was spread to approximately 12 mm diameter by a mirror and 2 inch diameter, 15-cm focal length N-BK7 plano-concave lens (Thorlabs Inc., Newton, NJ) on a 9-cm diameter petri dish containing 2 ml of MNV. The resulting intensity in this experiment was 0.15 W/cm^2^. All experiments assayed were performed in triplicate (*N* = 3) and each dilution was assayed in triplicate (*n* = 9). All sample temperature increases were < 3 °C above room temperature during all treatments.

### Assessment of RNA damage

RNA damage induced by the USPL was assessed by long RT-PCR using purified MNV RNA as described below. Two ml MNV samples were treated with 310 mW of fs 400 nm light for 2 and 4 h. Viral RNA was purified using the Viral amp kit (Qiagen) according to the manufactures instructions. Purified RNA template from treated and untreated samples was serially-diluted 10-fold and molecular amplification was performed as described by Kim and Ko [20] utilizing Qiagen one-step RT-PCR kit (Valencia, CA) in accordance with the procedures recommended by the manufacturer with 10 U of cloned RNAse inhibitor (Gibco-BRL) and forward 5′ ATG GTC CTG GAG AAT GGG TG 3′ and reverse primers 5’ TCC CGT AGA TCT TGT CTG GC 3′ to generate an 880 bp amplicon that was visualized by electrophoresis using a 1% agarose gel and ethidium bromide staining.

## Results

### MNV inactivation by variable wavelength visible fs light pulses and by CW

Table 1 shows that 400, 408, 425 and 450 nm femtosecond pulse laser light are all capable of nonthermally inactivating MNV, indicating that a discrete wavelength is not required for MNV inactivation. The ability of 408 nm CW light to inactivate MNV was also evaluated. As shown in Table 1, results indicate that 408 nm CW laser light does inactivate the virus, albeit less efficiently than the USPL since a 3 h-150 mW 408 CW treatment gave an average reduction of 0.82 log_10_, while the same treatment with the 408 fs USPL gave an average reduction of 3.39 log_10_.

**Table 1:** MNV inactivation by variable wavelength visible femtosecond and CW light. 1 ml samples were treated with 50 or 150 mW of USP laser or constant wavelength light configured as described in Fig. 1. (*N* = 3; *n* = 9)

Wavelength Laser treatments	Sample volume (ml)	Power (mW)	Light exposure time (h)	Log reduction	Std. Error
400 nm USP	1	50	4	1.43	± 0.47
425 nm USP	1	50	4	0.40	± 0.45
465 nm USP	1	50	4	0.12	± 0.12
510 nm USP	1	50	4	0.11	± 0.21
408 nm CW	1	150	3	0.82	± 0.12
408 nm USP	1	150	3	3.39	± 0.08
425 nm USP	1	150	3	3.24	± 0.10
450 nm USP	1	150	3	3.24	± 0.06

### Oxygen dependence

Inactivation of MNV by the USPL with variable wavelengths of blue light (400–450 nm) and by 408 nm CW indicated the inactivation mechanism was not specific to the 425 nm wavelength and, although more substantial inactivation was achieved with femtosecond pulse light, was not dependent on light pulses. This suggested that inactivation may have been via a non-ISRS mechanism. Given that the MNV samples tested are essentially complex mixtures of organic molecules resulting from viral lysis of host cells, centrifugation, and passage through a 0.2 μm filter, it is conceivable that singlet oxygen, or other reactive oxygen species, were produced directly by the lasers light’s interaction as a result of interplay between complex organic molecules present within the MNV sample and light.

Therefore two different experiments were performed to establish whether photochemistry could be the cause of virus inactivation. First, singlet oxygen generators, also called enhancers, were evaluated for the ability to enhance the inactivation observed in the presence of light. Rose bengal, methylene blue, and riboflavin, substances known to interact with light to generated singlet oxygen and other reactive oxygen species, were added to MNV samples. As shown in Table 2, experiments indicate that the enhancers in the presence of defocused 408 nm CW light do cause substantial inactivation, while defocused 408 CW light alone had a limited effect on MNV. Second, a 532 nm 7 ps laser light, a laser that does not inactivate MNV, was used to evaluate inactivation in the presence of singlet oxygen enhancers. Results are shown in Table 3 and demonstrate that the 532 laser substantially inactivates MNV when 0.1% rose bengal is present. Overall, it is evident that MNV is sensitive to singlet oxygen.

**Table 2:** Unfocused 408 nm CW with photosensitizers inactivates MNV

	Log reduction	Std. Error
Riboflavin		
Unfocused light	1.10	± 0.25
Rose Bengal		
Unfocused light	2.03	± 0.37
Methylene Blue		
Unfocused light	1.67	± 0.71
MNV w/o photosensitizer		
Unfocused light	0.07	± 0.02

As depicted in Fig. 1c, 1 h treatment of 2 ml of MNV with 0.1% rose bengal using 400 mw 408 nm defocused CW light laser light. (*N* = 3; *n* = 9)

**Table 3:** MNV is sensitive to photosensitizers: Treatment of 1 ml of MNV containing 0.1% rose bengal with a 100 mW picosecond 532 nm laser

	Log reduction	Std. Error
5 min	0.55	± 0.45
15 min	1.75	±0.38
30 min	2.63	± 0.17
1 h	3.77	± 0.25

Log reductions are based on untreated controls without rose bengal (*N* = 3; *n* = 9)

Given that dissolved oxygen within the MNV solution would theoretically be required for visible light to convert dissolved O_2_ to O_2_ (a^1^∆_g_) or other reactive oxygen species, experiments utilizing sodium bisulfite as an oxygen scavenger were performed. As noted in Table 4, addition of 200 ppm and 1000 ppm sodium bisulfite substantially reduced inactivation observed by the 408 CW laser.

**Table 4:** Oxygen sequestration: 3 h 450 mW CW 408 nm laser treatment of MNV in the presence and absence of sodium bisulfite

Sample	Sample volume (ml)	Power (mW)	Light exposure time (h)	Log reduction	Std. Error
MNV + 1000 ppm NaHSO_3_	1	450	3	1.35	± 0.51
MNV + 200 ppm NaHSO_3_	1	450	3	2.34	± 0.4
MNV w/o NaHSO_3_	1	450	3	3.40	± 0.67

Log reductions are calculated based on untreated MNV (*N* = 3; *n* = 9)

### Lack of MNV genome damage

**Fig. 2:**
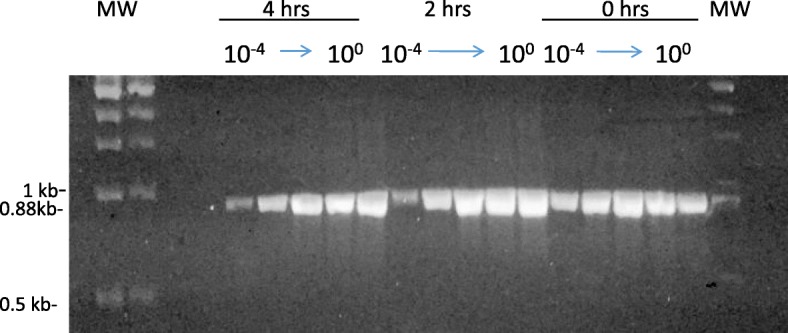
Viral RNA Damage Assessment. Two ml MNV samples were treated with the USPL at an average power of 310 mW for 0, 2, or 4 h. After MNV RNA, 1 μl of the 140 μl extracted, or serial ten-fold dilutions up to 10^− 6^ of that 1 μl sample were analyzed by RT-PCR. The 880 bp amplicon was visualized by electrophoresis using a 1% agarose gel. Dilutions up to 10^− 4^ are shown. No amplicon was observed for 10^− 5^ and 10^− 6^ samples

In order to test the effect of laser treatments on the MNV-1 RNA genome, RT-PCR was performed using 10-fold serially diluted RNA samples purified from USPL-treated virus samples. Visualization of 880 bp amplicons generated indicated that untreated, 2 and 4 h USP-treated samples gave identical amplifications, indicating that the viral RNA was not substantially damaged by the USPL treatments (Fig. 2).

## Discussion

Previous research indicated that a 425 nm USPL with an average power of 250 mW was able to non-thermally inactivate nonenveloped viruses and bacteriophages [42–46, 48, 49]. Essentially, vibrational resonance induced through an ISRS mechanism was hypothesized in which low frequency vibrational resonance modes of the virus capsid are induced that damage or destroy the virus capsid [43]. To test this hypothesis, experiments were carried out to investigate the dependence of pulse width and wavelength on virus inactivation, using a tunable USPL and a CW laser.

It was conceivable that wavelengths within the 420–430 bandwidth of the 425 nm laser light delivered in femtosecond pulses would be required to produce this virus inactivation effect. We report here that both a visible USPL ranging from 400 to 450 nm, and a CW laser (408 nm) are capable of inactivating MNV. We demonstrate that addition of photosensitizer molecules, known to generate singlet oxygen in the presence of light, greatly enhance inactivation of MNV, indicating that the virus is sensitive to singlet oxygen. This observation is essentially consistent with a recently published research investigation on the effects of 405 nm blue light on Tulane virus [19]. Further, the addition of sodium bisulfate, an oxygen scavenger, substantially reduced CW laser inactivation, strongly implicating singlet oxygen as the cause of visible laser light-induced virus inactivation.

Results of this study may conflict with a previous study which postulated that USPL induced impulsive stimulated Raman scattering (ISRS; [43] causing virus inactivation. The hypothetical ISRS inactivation mechanism would involve using a USPL to excite resonant vibrational modes capable of shaking the virus capsid apart. As the original laser used in the previous study was not tunable, it could not be determined whether observed inactivation was specific to 425 nm light, or was not specifically wavelength-dependent. Here, virus inactivation experiments were conducted with MNV using a wavelength-tunable USPL to demonstrate that inactivation is not wavelength-specific, since inactivation occurs with a range of USPL wavelengths between 400 and 450 nm. In these experiments, the optical band is approximately 10 nm in spectral width, approximately 5 nm above and below the tuned wavelength setting. ISRS theory dictates that the optical band must be broad enough to cover both a pump and Stokes wavelength, and the pulse width has to be shorter than the period of the vibrational mode to cause coherent excitement. Thus, a priori, one would not expect that vibrational resonance induced by ISRS to necessarily be wavelength-dependent. However, due to pulse-width dependence, it is well understood that ISRS cannot be induced using CW lasers since they do not generate light pulses. Here we clearly demonstrate that CW lasers can inactivate MNV, which potentially conflicts with the hypothesis that ISRS is the mechanism of inactivation as proposed by Tsen et al. [43] although it remains formally conceivable that both ISRS and singlet oxygen mechanisms could both contribute to laser inactivation observed by the USP laser. Furthermore, previous experiments indicated limited MNV inactivation when 425 nm USPL intensity was below 80 MW/cm^2^ [43]. Based on a focal length of 10 cm and a beam diameter of 0.1 cm, we estimate the average intensity of the CW laser at a maximum power of 500 mW to be only 24 KW/cm^2^ which is more than three orders of magnitude smaller than the previously observed 425 nm USPL threshold of 80 MW/cm^2^. Also, it is difficult to envision a scenario in which the low concentrations of sodium bisulfite that reacts with, and sequesters, dissolved oxygen molecules within the MNV sample would inhibit a laser-induced vibrational mechanism.

In the previous study using the USPL 425 nm laser [43], inactivation via a one photon absorption process [41] was discounted based on the lack of absorption of MNV at wavelength 425 nm and inactivation of a gradient centrifugation-purified virus sample which should have removed most extraneous cellular proteins and complex organic molecules that might absorb blue light and potentially act as singlet oxygen photoenhancers. Indeed it was noted that purified MNV and MNV from a cell lysate had roughly equivalent inactivation rates [43] suggesting that singlet oxygen enhancers are not substantially present in virus stocks, which are derived from virus-infected cell lysate. This suggests that either the virus capsid itself may function as an endogenous enhancer molecule, or that an enhancer may not be strictly required to produce singlet oxygen when interacting with intense blue light. To the latter point, published experiments with pressurized liquid oxygen identify several absorption peaks within the visible spectra that could directly excite oxygen [16] suggesting this may be possible.

Previously, Tsen et al. [43] noted highly damaged capsids via electron micrographs after 425 nm USPL treatments. It was suggested that hydrogen bonds and hydrophobic interactions critical for maintaining capsid integrity were overcome by laser-induced vibrational forces. However, these observations are also compatible with a mechanism involving photo-oxidation of amino acids such as cysteine, histidine, tyrosine, methionine and tryptophan within individual capsomer proteins [32]. Formation of peptide aggregates due to crosslinking induced by photo-oxidation chains has been described previously [7, 32]. Indeed when laser-treating mouse cytomegalovirus (MCMV), a herpesvirus, Tsen et al. [44] noted “formation of large strongly bound aggregates of MCMV capsid and matrix proteins that did not readily disassociate under dissociating or reducing conditions.” This observation would also be consistent with a photo-oxidative mechanism induced by reactive oxygen in which covalent crosslinks are formed between oxidized amino acids from adjacent polypeptide chains. Lack of demonstrable damage to viral RNA by the USPL is consistent with previous reports [42, 43, 48]. In H_2_O, the lifetime of singlet oxygen is short, estimated to be approximately 3.5 microseconds [51], providing limited time for diffusion into the virus capsid. For example, an estimate of O_2_ (a^1^∆_g_) diffusion produced intracellularly was estimated to be only 10 to 160 nm [33]. Thus singlet oxygen’s site of production must be very close to its site of action. Presumably, singlet oxygen generated outside the capsid would not have sufficient time to defuse within the virus capsid to substantially damage the viral RNA.

Overall the implication that reactive oxygen species are induced by light within the visible spectra has substantial bearing on the field of environmental virology, as well as food production. This finding offers a potential explanation as to why norovirus illness was originally termed the “winter vomiting disease.” It has been thought that cooler environmental temperatures permitted the virus to remain viable for longer periods [1, 26]. Results here would seem to support the idea that shorter, less-intense solar irradiation in the winter may also contribute substantially to environmental persistence of noroviruses.

## Conclusion

Results suggest formation of short-lived reactive oxygen species such as singlet oxygen by visible laser light as the cause of virus inactivation rather than via an ISRS mechanism which induces resonant vibrations.

## Abbreviations

bp: Base pairs
cm: Centimeter
CW: Continuous wave
EBSS: Earle’s balanced Salt solution
fs: Femtoseconds
ISRS: Impulsive stimulated Raman scattering
KW: Kilowatt
MCMV: Murine Cytomegalovirus
MNV: Murine norovirus
MW: Milliwatt
nm: Nanometer
nM: Nanomolar
pfu: Plaque-forming unit
ppm: Parts per million
ps: Picoseconds
RNA: Ribonucleic acid
RT-PCR: Reverse transcription-polymerase chain reaction
USP: Ultrashort pulse
USPL: Ultrashort pulse laser
W: Watts
μm: Micrometer

## References

[1] Ahmed SM (2013). Systematic review and meta-analysis of the global seasonality of norovirus. PLoSOne.

[2] Amin RM (2016). Antimicrobial blue light inactivation of *Pseudomonas aeruginosa* by photo-excitation of endogenous porphyrins: In vitro and in vivo studies. Lasers Surg Med.

[3] Bird RE (1983). Terrestrial solar spectral data sets. Sol Energy.

[4] Campos CJA, Lees DN (2014). Environmental transmission of human noroviruses in shellfish waters. Appl Environ Microbiol.

[5] Corbin F (2002). Pathogen inactivation of blood components: current status and introduction of an approach using riboflavin as a photosensitizer. Int J Hematol.

[6] Costa L (2012). Photodynamic inactivation of mammalian viruses and bacteriophages. Viruses.

[7] Davies MJ (2004). Reactive species formed on proteins exposed to singlet oxygen. Photochem Photobiol Sci.

[8] Felber TD et al: Photodynamic inactivation of herpes simplex: report of a clinical trial. J Am Med Assoc 1973, 92(3):223–289.

[9] Fisher MB (2014). Solar water disinfection (SODIS) of *Escherichia coli*, *Enterocoocus* spp. and MS-2 coliphage: effects of additives and alternative container materials. Water Res.

[10] Flannery JP (2013). Simulated sunlight inactivation of norovirus and fRNA bacteriophage in seawater. J Appl Microbiol.

[11] Fujioka RS, Yoneyama BS (2002). Sunlight inactivation of enteric viruses and fecal bacteria. Water Sci Technol.

[12] Goody RM, Yung YL (1989). Atmospheric radiation.

[13] Hall AJ (2013). Norovirus disease in the United States. Emerg Infect Dis.

[14] Heaselgrave W, Kilvington S (2012). The efficacy of simulated solar disinfection (SODIS) against coxsackie virus, poliovirus, and hepatitis a virus. J Water Health.

[15] Huang R (2004). Kinetics of singlet oxygen formation of riboflavin photosensitization and the reaction between riboflavin and singlet oxygen. J Food Sci.

[16] Jockusch S (2008). Singlet molecular oxygen by direct excitation. Photochem Photobiol Sci.

[17] Jori G (2006). Photodynamic therapy of microbial infections: state of the art and perspectives. J Environ Pathol Toxicol Oncol.

[18] Kingsley DH, Fratamico P, Kathariou S, Lui Y (2011). Food-borne Noroviruses. Genomes of Food- and Water-borne Pathogens.

[19] Kingsley DH (2018). Evaluation of 405 nm monochromatic light for inactivation of Tulane virus on the blueberry surfaces. J Appl Microbiol.

[20] Kim SY, Ko G (2012). Using propidium monoazide to distinguish between viable and nonviable bacteria, MS-2, and murine norovirus. Lett Appl Microbiol.

[21] Kohn T (2016). A modeling approach to estimate the solar disinfection of viral indicatior organisms in waste stabilization ponds and surface waters. Water Res.

[22] Kohn T, Nelson KL (2007). Sunlight-mediated inactivation of MS2 coliphage via exogenous singlet oxygen produced by sensitizers in natural waters. Environ Sci Technol.

[23] Lambrecht B (1991). Photoinactivation of viruses in human fresh plasma by phenothiazine dyes in combination with visible light. Vox Sang.

[24] Lenard J (1993). Photodynamic inactivation of infectivity of human immunodeficiency virus and other enveloped viruses using hypercin and rose bengal: inhibition of fusion and syncytia. Proc Natl Acad Sci.

[25] Liou KN (2002). An introduction to atmospheric radiation.

[26] Lopman B (2009). Host, weather and virological factors drive norovirus epidemiology: time-series analysis of laboratory surveillance data in England and Wales. PLoS One.

[27] Maclean M et al: A new proof of concept in bacterial reduction: antimicrobial action of violet-blue light (405 nm) in ex vivo stored plasma. J Blood Transfus 2016;2016:2920514. 10.1155/2016/2920514 PMC5059568 27774337

[28] Maclean M (2009). Inactivation of bacterial pathogens following exposure to light from a 405-nanometer light-emitting diode array. Appl Environ Microbiol.

[29] Matthews JL (1992). Inactivation of viruses with photoactive compounds. Blood Cell.

[30] Mirshafiee H (2015). The effects of ultraviolet light and riboflavin on inactivation of viruses and the quality of platelet concentrates at laboratory scale. Avicenna J Med Biotechnol.

[31] Mohr H (1997). Virus inactivation of blood products by phenothiazine and light. Photochem Photobiol.

[32] Nilsson R (1972). Unambiguous evidence for the participation of singlet oxygen in photodynamic oxidation of amino acids. Photochem Photobiol.

[33] Ogilby PR (2010). Singlet oxygen: there is indeed something new under the sun. Chem Soc Rev.

[34] Perdrau JR, Todd C (1933). Photodynamic action of methylene blue on certain viruses. Pro Roy Soc Lond B Biol Med.

[35] Rosado-Lausell SL (2013). Roles of singlet oxygen and triplet state of dissolved organic matter formed by different organic matters in bacteriophage MS2 inactivation. Water Res.

[36] Scallan E (2011). Foodborne illness acquired in the United States--major pathogens. Emerg Infect Dis.

[37] Silverman AI (2013). Sunlight inactivation of human viruses and bacteriophages in coastal waters containing natural photosensitizers. Environ Sci Technol.

[38] Sinton LW (1999). Sunlight inactivation of fecal bacteriophages and bacteria in sewage polluted seawater. Appl Environ Microbiol.

[39] Skwor TA (2016). Photodynamic inactivation of methicillin-resistant *Staphylococcus aureus* and *Escherichia coli*: a metalloporphyrin comparison. J Photochem Photobiol B.

[40] Sloand EM (1995). Safety of the blood supply. J Am Med Assoc.

[41] Tkachenko NV. Appendix C. Two photon absorption. In: Optical spectroscopy: methods and instrumentations. Oxford: Elsevier; 2006. p. 293.

[42] Tsen SW (2012). Inactivation of enveloped virus by laser-driven protein aggregation. J Biomed Optics.

[43] Tsen SW (2014). Studies of inactivation mechanism of non-enveloped icosahedral viruses by a visible ultrashort pulsed laser. Virology J.

[44] Tsen SW (2014). Pathogen reduction in human plasma using an ultrashort pulsed laser. PLoS One.

[45] Tsen KT (2007). Inactivation of viruses by coherent excitations with a low power visible femtosecond laser. Virology J.

[46] Tsen KT (2007). Inactivation of viruses by laser-driven coherent excitations via impulsive stimulated Raman scattering process. J Biomedical Optics.

[47] Tsen KT (2007). Inactivation of viruses with a very low power visible femtosecond laser. J Phys: Condensed Matter.

[48] Tsen KT (2009). Photonic approach to the selective inactivation of viruses with a near-infrared subpicosecond fiber laser. J Biomed Optics.

[49] Tsen KT (2011). Studies of inactivation of encephalomyocarditis virus, M13 bacteriophage, and *Salmonella typhimurium* by using a visible femtosecond laser: insight into the possible inactivation mechanisms. J Biomed Opt.

[50] Wainright M (2000). Methylene blue derivatives-suitable photoantimicrobial for blood product disinfection?. Int J Antimicro Ag.

[51] Wilkinson F (1995). Rate constants for the decay and reactions of the lowest electronically excited singlet-state of molecular-oxygen in solution—an expanded and revised compilation. J Phys Chem Ref Data.

[52] Zahorsky J (1929). *Hyperemisishiemis* or the winter vomiting disease. Arch Pediatr.

